# Resin-Loaded Heterogeneous Polyether Sulfone Ion Exchange Membranes for Saline Groundwater Treatment

**DOI:** 10.3390/membranes12080736

**Published:** 2022-07-27

**Authors:** Fulufhelo Mudau, Machawe Motsa, Francis Hassard, Lueta-Ann de Kock

**Affiliations:** 1 Institute for Nanotechnology and Water Sustainability, College of Science, Engineering and Technology, University of South Africa, Johannesburg 1709, South Africa; fulufhelohope86@gmail.com (F.M.); motsamm@unisa.ac.za (M.M.); 2 Cranfield Water Science Institute, Cranfield University, College Way, Bedford MK43 0AL, UK; francis.hassard@cranfield.ac.uk

**Keywords:** heterogeneous ion exchange membranes, ultrafiltration, ion exchange resin loading, ion exchange capacity, groundwater treatment, salt rejections

## Abstract

Arid areas often contain brackish groundwater that has a salinity exceeding 500 mg/L. This poses several challenges to the users of the water such as a salty taste and damage to household appliances. Desalination can be one of the key solutions to significantly lower the salinity and solute content of the water. However, the technology requires high energy inputs as well as managing waste products. This paper presents the fabrication of ultrafiltration heterogeneous ion exchange membranes for brackish groundwater treatment. Scanning electron microscopy (SEM) images showed a relatively uniform resin particle distribution within the polymer matrix. The mean roughness of the cation exchange membrane (CEM) and anion exchange membrane (AEM) surfaces increased from 42.12 to 317.25 and 68.56 to 295.95 nm, respectively, when resin loading was increased from 1 to 3.5 wt %. Contact angle measures suggested a more hydrophilic surface (86.13 to 76.26° and 88.10 to 74.47° for CEM and AEM, respectively) was achieved with greater resin loading rates. The ion exchange capacity (IEC) of the prepared membranes was assessed using synthetic groundwater in a dead-end filtration system and removal efficiency of K^+^, Mg^2+^, and Ca^2+^ were 56.0, 93.5, and 85.4%, respectively, for CEM with the highest resin loading. Additionally, the anion, NO_3_^−^ and SO_4_^2−^ removal efficiency was 84.2% and 52.4%, respectively, for the AEM with the highest resin loading. This work demonstrates that the prepared ultrafiltration heterogeneous ion exchange membranes have potential for selective removal for of ions by ion exchange, under filtration conditions at low pressure of 0.05 MPa.

## 1. Introduction

Groundwater often serves as an alternative potable water source for arid environments. Normally, this water is fit for direct use and is often characterised by hardness due to the presence of calcium and magnesium salts, which gives it a bad taste. However, the total dissolved solids and electrical conductivity of the water would still be within the World Health Organization (WHO) limits for drinking water. However, recently, there has been an increase in the salt content of groundwater due to natural processes and human influences. Extreme increase in ionic strength may be attributed to seawater infiltration, poor irrigation practices, or groundwater over-abstraction, which result in salt concentration in aquifers [1]. Consequently, groundwater often exceeds the World Health Organization (WHO) guidelines for drinking water, and key constituents that impact health, nutrition, and aesthetics of the water are affected [2]. Major constituents of concern for drinking water with significant health effects are nitrates and hardness, and more often than not, groundwater chemistry is determined by geological strata [3]. High salt concentrations are commonly associated with arid/semi-arid climates, low surface water availability, and advanced stages of groundwater abstraction [4,5,6]. For example, in South Africa, groundwater contributes 15% of the total water resources used, with the majority used for agricultural irrigation (60%) [7]. This does not include other uses such as domestic, mining, and industry. Part of the allure of groundwater sources is that they typically require less treatment than most surface waters and can be available even during dry periods or in environments with highly variable source quality [3]. However, the over-abstraction of groundwater leads to high salt levels. The increase in salt concentration occurs when groundwater moves through sedimentary rocks and soils, picking natural occurring elements such as magnesium, calcium, chloride, arsenate, fluoride, nitrate, and iron at unacceptable drinking water levels [8]. Hence, there is a need for groundwater treatment before potable use.

Desalinating technologies such as reverse osmosis (RO) membranes are ideal for purifying saline groundwater as they reject di- and monovalent ions as well as micro/trace organics [9,10]. The main reason behind this prolific rejection is the dense selective layer that is semipermeable and requires extensive external hydraulic pressure to drive water through the membrane. In addition, whilst RO is highly effective at removing salts from water, it produces a waste concentrate with potential toxicity that presents a health and environmental hazard if not treated or disposed of safely [11]. Inland RO concentrate disposal is a challenge, and current options include surface water discharge, sewer discharge, deep well injection, evaporation ponds, irrigation, and zero liquid discharge using thermal evaporators and crystallisers [1]. Although there are disposal options inland, disposal of large amounts of RO concentrate in inland communities can be cost-prohibitive, and over time, the requirements for concentrate disposal become stricter. Therefore, developing new methods that reduce concentrate waste is crucial to keeping costs and environmental damage down.

Several desalination technologies have been evaluated to improve concentrate management and recovery, and this includes dew-vaporation, membrane distillation, forward osmosis, electrodialysis, electrodialysis reversal, electrodialysis metathesis, and various intermediate precipitation followed by secondary RO processes [1]. These technologies have excellent water recovery (90%) but are often costly and energy-extensive. A cost-effective alternative is required to selectively remove specific constitutes (e.g., nitrates, phosphates, monovalent ions) in brackish water which partially reduce the salt levels and meet water quality requirements for a target application. Ion exchange membrane technologies are a commonly used method for water desalination whereby semi-permeable membranes provide ionic selectivity due to fixed charged functional groups on the membrane surface [12]. Ion exchange membranes are classified according to the fixed functional groups into cation exchange and anion exchange membranes [13,14]. Cation exchange membranes with negatively charged groups allow the transportation of cations while rejecting anions. On the other hand, anion exchange membranes have positively charged groups that allow anions to pass and reject cations [13,15,16]. Ion exchange membranes are either homogeneous, where the charged groups are chemically bonded to the membrane matrix, or heterogeneous, where ion exchange particles are physically mixed with the membrane matrix [17]. In terms of functionality, a trade-off between the two types of membranes exists. Homogeneous ion exchange membranes have good electrochemical properties but poor mechanical strength, whereas heterogeneous ion exchange membranes possess acceptable mechanical properties but have comparatively poor electrochemical properties [18,19].

Even though heterogeneous membranes have comparatively poor electrochemical properties, they provide advantages such as low cost, ease of manufacturing, favourable oxidative stability, and good mechanical strength [15,20,21]. Heterogeneous ion exchange membranes’ properties can be tailored by varying the nature of functional groups, selection of polymeric matrices, degree of crosslinking, nature of the surface layer, and variation of heterogeneity [22]. Additionally, the use of suitable binders makes it possible to produce ion exchange membranes with an optimal combination of electrochemical and mechanical strength [15]. However, attaining one desirable property of an ion exchange membrane is usually at the expense of another parameter. Optimisation of variables is necessary to obtain membranes with characteristics suitable for the specific requirements of the application [23].

The fixed charged functional groups of the ion exchange membranes determine most properties of the membrane such as water uptake, selectivity, and electrical resistance [24]. To increase the selectivity of a membrane towards specific ions (mono- or bivalent ions), there are some possibilities to adjust the interaction between ions and membrane [25]. The selectivity is largely determined by the ion exchange resin structure, and naturally, different ions are selected according to the charge number [26]. The selective behaviour of an ion exchange membrane involves optimising the ion transport number. One possible route to enhance transport number is to have a heterogeneous membrane with a homogenised surface, which will enhance the selectivity of specific ion type [27]. Another approach is to increase the ion exchange resin content inside the membrane matrix. Previous studies have shown that increasing the ion exchange resin content increases the transport number of ions making them more selective toward specific ions, but this may compromise membrane mechanical strength [19,28,29].

Here, ultrafiltration heterogeneous ion exchange membranes were prepared by adding novel amounts of ion exchange resins onto the membrane matrix to achieve the desired structural integrity for ion removal and macromolecules retention application. The macromolecules and dissolved organic matter are retained through size exclusion and surface interactions, whilst the resins selectively remove ionic species (anion and cations). The ion exchange membranes are based on polyethersulfone (PES) acting as a binder for ion exchange resin particles with a pore former, polyethylene glycol (PEG), which aided uniform pore size and distribution through the sub-surficial layer of the membrane. Thus, it is hypothesised that ion exchange resin loading during fabrication would change the membrane morphology by providing selective water channels presented by the resins. Additionally, improved porosity through the creation of pores and voids at the polymer-resin interface. Membrane porosity will improve water permeation and subsequently lower the applied driving pressure.

## 2. Materials and Methods

### 2.1. Materials

Polyethersulfone (PES) (Sigma Aldrich, St. Louis, MI, USA) was used as a polymer binder and Polyethylene glycol 10,000 (PEG) (Sigma Aldrich) as a pore former. 1-Methyl-2-pyrrolidone (NMP) (Sigma Aldrich) was employed as a solvent. A strong acidic cation exchange resin (Amberlyst 15) and a strong basic anion exchange resin (Amberlite IRA 900) were purchased from Sigma Aldrich. A microfiltration polyester non-woven fabric was purchased from Hirose Paper Manufacturing Co. Ltd. in Tosa-shi, Japan. The synthetic groundwater solution was prepared by dissolving potassium nitrate (KNO_3_, Sigma Aldrich), magnesium sulfate (MgSO_4_, Sigma Aldrich), calcium chloride (CaCl_2_, Sigma Aldrich), sodium nitrate (NaNO_3_, Sigma Aldrich), sodium bicarbonate (NaHCO_3_, Sigma Aldrich) and potassium bicarbonate (KHCO_3_, Sigma Aldrich) in ultrapure water. MERCK Certipur 111,355 ICP multi-element standard solution IV was used to prepare external standards for inductively coupled plasma atomic emission spectrometry (700 Series ICP-OES, Agilent Technologies, Santa Clara, CA, USA). Potassium cell test (K^+^ cat. No: 1.14562), sulfate cell test (SO_4_^2−^, cat. No: 1.02537), chloride cell test (Cl^−^, cat No: 114730), total hardness test (cat. No: 1.00961) and nitrate cell test (NO_3_^−^ cat. No: 1.14773) were purchased from Merck (Darmstadt, Germany).

### 2.2. Preparation of Heterogeneous Cation and Anion Exchange Membranes

The heterogeneous ion exchange membranes were prepared by the casting solution technique and phase inversion method. Amberlyst 15 (strong acid cation exchange resin, sulfonic acid functional group) and Amberlite IRA 900 (strong base anion exchange resins, quaternary ammonium functional group) were selected to provide functional groups for the membrane. Ion exchange resins (Amberlyst 15 and Amberlite IRA 900) were pulverised using a mortar and pestle into fine particles and sieved to the desired mesh size (≤100 µm). PES (15 wt %), PEG (2 wt %), and ion exchange resin powder were mixed with NMP solvent in a glass reactor equipped with a mechanical stirrer overnight at 50 °C. Vigorous mixing was necessary to obtain uniform particle distribution in the polymeric solution. The mixture was then cast onto a polyester non-woven fabric attached to a clean and dry glass plate using a manual casting knife with a gap height of 150 µm. The glass was immediately immersed into a bath of deionised water for 24 h. The membranes were subsequently immersed in 0.1 M HCl for 24 h, and stored in 0.5 M NaCl until they were used. Table 1 below shows the casting mixture composition. The ion exchange resin powder loading was kept low up to 3.5 wt % in the casting solution because beyond 3.5 wt % loading, the membrane structural integrity becomes compromised (i.e., brittle structure with defects on the surface).

### 2.3. Membrane Characterisation

#### 2.3.1. Membrane Morphology and Topography

Scanning electron microscope (SEM) (JOEL IT 300 SEM, Tokyo, Japan) was used to study the morphology of the membrane surfaces. Samples for cross-sectional images were obtained by breaking the membrane samples under liquid N_2_. All samples were coated with gold (Quorum Q150R ES coater, Quorum Technologies, Laughton, UK) before SEM imaging. Microscopic observation of surface topology and roughness of the prepared membranes was performed by an Atomic Force Microscopy (AFM) (Alpha300, WITec, Ulm, Germany). Small squares of the prepared membranes (approximately 1 cm^2^) were cut and attached on a glass substrate for analysis. The membrane surfaces were imaged using a scan size of 50 µm × 50 µm.

#### 2.3.2. Hydrophilicity

Membrane surface wettability and, subsequently, its hydrophilicity, was studied using contact angle measurements using a Drop shape analyser (Kruss, Hamburg, Germany). Contact angles were measured at more than 10 random locations across the membrane surface for each sample and the average value reported.

#### 2.3.3. Water Uptake

Water uptake was determined by the weight difference between dried and wet membrane samples. Dry membrane samples (1 cm by 1 cm) were immersed in distilled water for 24 h, excess water was wiped from the surface, then the samples were immediately weighed (RADWAG, Model: AS 82/220.R2). The weight of the same piece was recorded after being dried in the oven for 4 h at 60 °C. The percentage of *water uptake* was calculated by Equation (1):

(1) $$Water\;uptake\;\left(\%\right)=\frac{W_{wet}-W_{dry}}{W_{wet}}\times 100$$

where *W_wet_* (g) is the weight of the wet membrane and *W_dry_* (g) is the weight of the dry membrane. Percentage water uptake was determined three times for each sample and the average value was reported.

#### 2.3.4. Ion Exchange Capacity (IEC) and Fixed Ion Concentration (FIC)

The ion exchange capacity (IEC) of the membranes was determined by a titration method [18,30,31]. The cation exchange membrane samples (1 cm by 1 cm) were initially charged with H^+^ ions by soaking in 1.0 M HCl solution for 24 h. The membranes were rinsed several times with deionised water, then immersed in a 2.0 M NaCl solution (20 mL) for another 24 h to replace H^+^ ions with Na^+^. On removing the membrane, the NaCl solution was titrated with 0.01 M NaOH using phenolphthalein as an indicator. The *IEC* was calculated using Equation (2).

(2) $$IEC\left(\mathrm{meq}\cdot g^{-1}\right)=\left(\frac{a}{W_{dry}}\right)$$

where *a* is the milli-equivalent of the ion exchange group in membrane and *W_dry_* is the weight of dry membrane.

A similar procedure was followed for the anion exchange membranes. The membrane samples were soaked in 1 M HCl solution for 24 h to convert the anion exchange functional groups in the membrane to the Cl^−^ form. The membrane samples were placed in 2 M NaCl solution for another 24 h and washed with water until there was no visible white residue. The membranes were then placed into 2 M NaNO_3_ solution for another 24 h to release the Cl^−^ into solution. The solution containing Cl^−^ was titrated with 0.01 M AgNO_3_ using K_2_CrO_4_ as an indicator. The *IEC* was calculated as expressed in Equation (2).

Fixed ion concentration is the relationship between the *IEC* and water content. The *FIC* can be calculated by:

(3) $$FIC\left(\mathrm{meq}\cdot g^{-1}\right)=\left(\frac{IEC}{Water\;content}\right)$$

#### 2.3.5. Membrane Potential, Transport Number and Permselectivity

**Figure 1:**
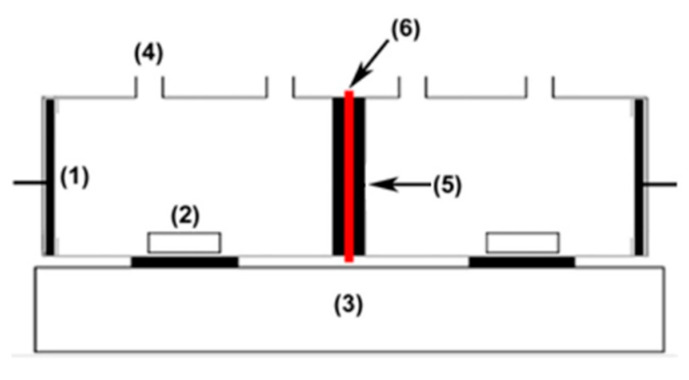
Schematic diagram of testing apparatus for membrane potential: (1) Pt electrode, (2) magnetic bar, (3) stirrer, (4) orifice, (5) rubber ring, (6) membrane.

Membrane potential is the algebraic sum of the Donnan and diffusion potentials determined by the partitioning of ions into the pores as well as the mobilities of ions within the membrane phase compared to the external phase. Membrane potential was evaluated for the equilibrated membrane with unequal concentrations (C_1_ = 0.1 and C_2_ = 0.01 M) of sodium chloride solution at ambient temperature on either side of the membrane sample using a two-cell glassy apparatus (Figure 1). The solutions in both sections were stirred vigorously to minimise the effect of boundary layers. The potential that developed across the membrane was measured by connecting both compartments using standard electrodes and a digital automatic multi-meter (EX210T: True RMS mini multimeter with IR, Extech Instruments, Nashau, NH, USA). The measurement was repeated until a constant membrane potential value was obtained. Membrane potential (*E_Measure_*) is determined using the Nernst Equation (4):

(4) $$E_{Measure}\left(\mathrm{mV}\right)=\left(2t_{i}^{m}-1\right)\left(\frac{RT}{nF}\right)ln\left(\frac{a_{1}}{a_{2}}\right)$$

where *t_i_^m^* is the transport number of counter ions in membrane phase, *R* (8.3145 J mol^−1^ K^−1^) is the universal gas constant, *T* (K) is the temperature, *n* is the electrovalence of the counter ion, *F* (96.5 KJ mol^−1^) is the Faraday constant, and *a*_1_ and *a*_2_ are electrolyte activities of solutions in contact with the membrane surfaces.

The measured membrane potential is used to calculate the transport number ($t_{i}^{m})$ of the counter ions in the membrane phase by re-arranging the Nernst equation and using Equation (5) to determine $t_{i}^{m}$:

(5) $$t_{i}^{m}=\frac{E_{measure}}{2\left(\frac{RT}{nF}\right)ln\left(\frac{a_{1}}{a_{2}}\right)}+1$$

The ionic permselectivity of membranes can be quantified based on the migration of counter-ions through the IEMs (Equation (6)).

(6) $$P_{s}=\left(\frac{t_{i}^{m}-t_{0}}{1-t_{0}}\right)$$

where *t*_0_ is the transport number of counter ions in the solution.

### 2.4. Membrane Testing and Application

Pure water flux and salt rejection experiments were conducted using a dead-end cell with an effective membrane area of 0.0128 m^2^. Each of the membranes was pre-compacted by filtration of water at a pressure of 0.10 MPa for at least 40 min, followed by applying the operating pressure of 0.05 MPa. The pure water flux was measured until stable water flux (*J_w_*_1_) was achieved (Equation (7)). Each experiment was performed in triplicate, and the average was reported.

(7) $$J_{w1}=\frac{Q}{A\Delta t}$$

where *J_w_*_1_ represents water flux (L·m^−2^·h^−1^), *Q* represents water permeate volume (L), *A* represents membrane area (m^2^) and Δ*t* represents permeation time (h).

Synthetic groundwater was prepared in the laboratory and used for ion rejection studies. The Idaho National Laboratory synthetic groundwater recipe (based on chemical analysis of groundwater samples collected at the Idaho National Laboratory Site) was used. The synthetic groundwater contained 4 mg/L KNO_3_, 110 mg/L MgSO_4_, 194 mg/L CaCl_2_, 3.4 mg/L NaNO_3_, 92.4 mg/L NaHCO_3_, 6.2 mg/L KHCO_3_ at pH 7.06 and total dissolved solids (TDS) of 410 mg/L. The rejection tests were performed at 0.05 MPa pressure with a sample of permeate solution collected every 30 min. A pressure of 0.05 MPa was used to increase the residence time and to enable equilibration of ion exchange to occur. The concentrations of Na^+^, K^+^, Ca^2+^, Mg^2+^, SO_4_^2−^, NO_3_^−^, HCO_3_^−^ and Cl^−^ in the feed and permeate solutions for each of the membranes was determined. The concentrations of Na^+^, Ca^2+^ and Mg^2+^ ions were determined by ICP-OES) (Agilent Technologies 700 Series ICP-OES). Concentrations of K^+^, SO_4_^2−^, Cl^−^, HCO_3_^−^ and NO_3_^−^ ions were determined photometrically (Spectroquant Pharo 300, Merck, Darmstadt, Germany). The salt rejection percentage (SR %) was obtained as follows (Equation (8)):

(8) $$R\left(\%\right)=\frac{C_{f}-C_{p}}{C_{f}}\times 100$$

where *C_f_* (mg/L) represents the concentration of the feed solution and *C_p_* (mg/L) is the concentration of permeate.

## 3. Results and Discussion

### 3.1. Membrane Characterisation

#### 3.1.1. Membrane Morphology

**Figure 2:**
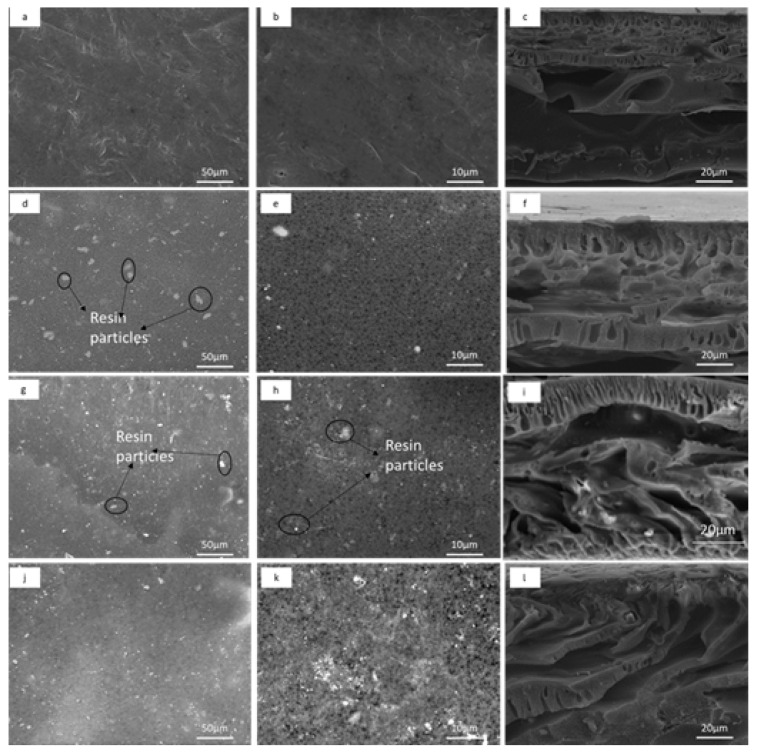
SEM images of bare PES and heterogeneous CEMs: (**a**) surface images of bare PES at ×500, (**b**) surface images of bare PES at ×1000, (**c**) cross-sectional images of bare PES, (**d**) surface images of CEM-1 at ×500, (**e**) surface images of CEM-1 at ×1000, (**f**) cross-sectional images of CEM-1, (**g**) surface images of CEM-2.5 at ×500, (**h**) surface images of CEM-2.5 at ×1000, (**i**) cross-sectional images of CEM-2.5, (**j**) surface images of CEM-1 at ×500, (**k**) surface images of CEM-3.5 at ×1000, and (**l**) cross-sectional images of CEM-3.5.

**Figure 3:**
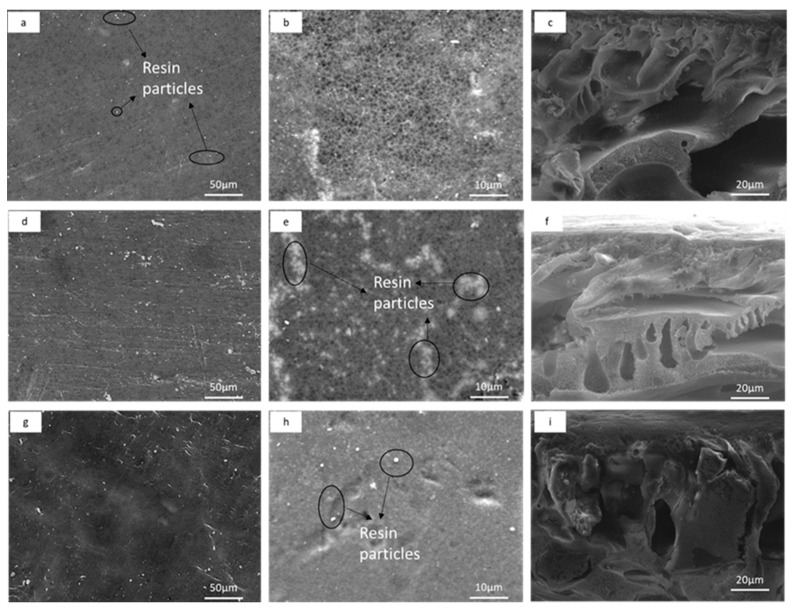
SEM images of custom-made UF heterogeneous AEMs: (**a**) surface images of AEM-1 at ×500, (**b**) surface images of AEM-1 at ×1000, (**c**) cross-sectional images of AEM-1, (**d**) surface images of AEM-2.5 at ×500, (**e**) surface images of AEM-2.5 at ×1000, (**f**) cross-sectional images of AEM-2.5, (**g**) surface images of AEM-3.5 at ×500, (**h**) surface images of AEM-3.5 at ×1000, and (**i**) cross-section images of AEM-3.5.

The SEM images of the bare PES membrane, CEMs and AEMs at varying resin content are shown in Figure 2 and Figure 3. The bare PES membrane surface exhibited a uniform, continuous structure (Figure 2a). The CEMs surface images show the presence of uniformly distributed cation exchange resin particles (white spots) which are embedded in the PES polymer binder (darker areas) matrix (Figure 2d–k). Increasing the resin particles loading from 1 to 3.5 wt % in the dope solution led to more resin particles (conducting regions) per unit area (Figure 2e,h,k). At a higher SEM image resolution, the surface morphology changed with resin loading (Figure 2e,h,k). Cross-sectional morphology revealed that it almost remained constant with only the selective layer undergoing some changes. The cross-section images of 1% resin loading (CEM-1, f) had a symmetrical configuration with layers of macrovoids distributed on both the upper and lower layers. Increasing the resin loading beyond 1% reverted the structure to an asymmetric one, with two different layers. The upper layer of CEM-2.5 and CEM-3.5 appears to be a very dense layer (selective layer), while the morphology of the lower layer is a combination of finger-like and spongy structures (Figure 2i,l).

The surface and cross-section SEM images of the prepared AEMs are shown in Figure 3. As previously seen with the CEMs, the surface SEM images also showed similar morphology. The anion exchange resin particles were uniformly distributed and embedded in the PES polymer binder matrix (Figure 3a–h). As with CEM membranes, the inclusion of anion exchange resins in the membrane matrix changed the surface morphology resulting in a dense membrane. The cross-sectional images of AEMs indicated a dense upper layer (selective layer) and spongy sub-layer with macrovoids (Figure 3c,f,i). The SEM images also showed more resin particles per unit area, with increased resin loading in the dope solution, as with the CEMs.

The ion exchange membrane morphology associated with resin particles content and distribution within the membrane is a critical parameter for the membrane electrochemical performance [28]. Khodabakhshi et al. reported that the uniform distribution and presence of more ion exchange resin particles per unit area provides superior conducting regions in the membrane and easy flow for the transportation of counter-ions [32]. Several heterogeneous IEMs have been previously prepared with the aim of treating water, and the average resin loading has been 50 wt %, which resulted in a homogeneous distribution of resin particles within the polymer binder and a decreased length of the inert regions (polymer binder) between resin particles. However, an increased resin loading of 45 to 70 wt % results in fractures on the membrane surface [33]. These fractures occur due to unstable dope solution which subsequently result in weak and poor membranes [34]. In this work, the resin loading rate was kept low up to 3.5 wt % to allow for a mixed matrix system to have uniform porosity that allows both filtration and ion exchange simultaneously. The prepared IEMs and bare PES membrane were porous, as indicated by the SEM images (Figure 2 and Figure 3). This is attributed to the addition of a pore former PEG in the casting solution [35]. This is a desired structural property for the proposed filtration and ion exchange application.

#### 3.1.2. Membrane Topography

The surface roughness of the bare PES membrane, CEMs and AEMs was studied using AFM analysis (Table 2) and assessed by the topography parameters: Sa represents the mean surface roughness of the sample, and Sq is the root mean square roughness. The three-dimensional AFM images of the membrane surfaces of PES, CEM and AEM are available in Supplementary Materials Figure S1. The bare PES membrane gave significantly higher Sa values than the CEMs and AEMs. These results indicated that the bare PES membrane surface is rougher than the prepared CEMs and AEMs (Table 2). For CEM and AEM membranes, Sa values increased with increasing resin loading from 1 to 3.5 wt %. The resin particles in CEMs and AEMs matrix resulted in inhomogeneity on the membrane surface (Figure S1). Thus, resin particles and PES have separate phases which form peaks and valleys on the membrane surface and surface roughness. The surface roughness of the prepared bare PES membrane, CEMs and AEMs is also related to membrane porosity due to the addition of PEG as a pore former [36]. Thus, the depressions and high peaks (nodules) formed during the polymer coagulation process are responsible for the increased surface roughness [37]. The immersion of the casting solution in a coagulation bath causes variation in the composition of the mixture, which makes the homogeneous casting solution thermodynamically unstable [38]. The instability causes the solution to decrease its free energy of mixing by dividing into two liquid phases of different compositions, i.e., the nucleation of polymer-poor phase (PEG) forms the depressions, and the polymer-rich phase (PES) surrounds the depressions [39]. In this work, the CEMs and AEMs are less rough than the bare PES because resin particles are polymer-poor and accumulate in the depressions, which smoothed the membrane surface [40]. The AEM-1 (68.56 nm) and AEM-2.5 (153.41 nm) membrane surfaces are rougher than the CEM-1 (42.12 nm) and CEM-2.5 (102.08 nm) membranes according to the Sa values (Table 2). The possible reason for the differences in morphology is due to the interaction between the sulfonic acid group of the cation exchange resin and the PES polymer binder (i.e., electrophilic aromatic substitution type interactions, wherein the hydrogen atom in the ortho position of aromatic PES polymeric chain can be replaced by a sulfonic group of the cation exchange resin) [41,42]. Therefore, during phase separation, the cation exchange resin particles distribute themselves more within the membrane matrix (polymer-rich phase) because of the interactions. However, the anion exchange resins with no interactions mostly separate themselves from the polymer-rich phase of the membrane, resulting in more anion exchange membrane filling the pores and making the AEMs’ surfaces rougher [38].

#### 3.1.3. Hydrophilicity

The measured contact angles for the bare PES membrane, CEMs and AEMs are presented in Table 2. The contact angle of the bare PES membrane was 88.89 degrees, making it hydrophobic. It is expected of the PES membrane since PES is known to be a slightly hydrophobic polymer [43]. The contact angles of the CEMs and AEMs were lower than that of the bare PES membrane. Additionally, CEMs and AEMs contact angles decrease with increasing resin loading (from 1 to 3 wt %), which means membrane surface hydrophilicity increased with resin loading. This effect is due to the sulfonic acid groups and quaternary amine groups present in the cation and anion exchange groups of the ion exchange resin particles that make up the CEMs and AEMs. The presence of charged functional groups results in hydrogen bonding with water molecules, which improves membrane hydrophilicity [28]. These functional groups become hydrated, resulting in membrane swelling [18,31,36]. The PEG additive (pore former) in the casting solution also improves membrane surface hydrophilicity of the bare PES membrane, CEMs and AEMs. Ma et al. reported that PEG, being hydrophilic in nature, is used for the suppression of microvoids and to give the membrane a hydrophilic character. The surface hydrophilicity of CEM and AEM is due to hydrophilic PEG additive and hydrophilic ion exchange resin particles. The contact angles values (Table 2) indicated that AEMs are more hydrophilic than CEMs. Additionally, the results are attributed to the coverage of cation exchange resin by the hydrophobic PES polymer chains due to the interactions between them (see section membrane topography discussion section), which reduce the accessibility of hydrophilic resin particles [44].

#### 3.1.4. Water Uptake

Water uptake measurements of the bare PES membrane, CEMs and AEMs are presented in Table 2. The bare PES membranes’ water uptake value is 58.89%, making it lower than the CEMs and AEMs. The water uptake values of the CEMs slightly increased from 61.20% to 65.55% as resin loading increased. The AEMs resin loading did not impact the water uptake since the water uptake remained at 63% regardless of the resin loading. The water uptake of the bare PES membrane, CEMs and AEMs is attributed to macrovoids observed in the SEM cross-sectional images (Figure 1 and Figure 2). Thus, the presence of macrovoids within the membrane matrix results in more intestinal volume to take up water [23,31]. According to Hosseini et al. (2012), the addition of PEG into the casting solution results in the formation of macrovoids (voids and cavities), which causes the membrane to accommodate more water molecules. The water uptake value of CEMs and AEMs was higher than the bare PES membrane, which is attributed to the PES polymer hydrophobic nature. Additionally, the inclusion of resin particles in membrane matrix bring discontinuity in the polymer and more water pockets are created. Thus, the number of macrovoids present in the CEMs and AEMs is higher the number for the bare PES due to increased microvoids. The AEMs water uptake values were lower than the CEM because of the difference in ion exchange resins used in membrane fabrication. This observation is due to functional groups of AEM having no interactions with the PES, which results in the anion exchange resin particles distributing themselves in the membrane pores (polymer-poor phase) after the coagulation process, which reduces the water uptake. On the other hand, cation exchange resin has electrophilic substitution interactions with PES polymer, resulting in some of the cationic resin being covered by the PES polymer matrix, which creates additional microvoids within the membrane matrix [42]. The water uptake is higher than those reported in the previous literature, and the reported water uptake of heterogeneous ion exchange membranes is between 20 and 40% [45]. In this study, the water uptake was above 50%, which may be attributed to the low resin content (1 to 3.5 wt %). Jashni et al. (2019) reported that a high concentration of resin content reduces water accommodation in the membrane matrix, which reduces water uptake and previous studies had a resin loading of 40–60% [22,46,47]. The high water uptake obtained is a desired feature for filtration, and it provides more and wider transfer channels for the transportation of co and counter ions [18].

#### 3.1.5. Ion Exchange Capacity and Fixed Ion Concentration

Ion exchange capacity (IEC) is a measure of the ionic conductivity of membranes (Kimberly et al., 2018). The bare PES membrane had no IEC, and the IEC values for CEMs and AEMs increased from 0.06 to 0.58 meq/g and from 0.5 to 1 meq/g, respectively, with increasing resin loading from 1 to 3.5 wt % (Table 3). The increasing IEC is attributed to the increasing number of ion exchange resin particles per unit area of the membrane matrix. The respective sulfonic acid group and quaternary amine groups of the cation and anion exchange resins enhance the polar–polar interactions between the membrane surface and solution phase [31]. These polar–polar interactions facilitate the transportation of ions between the membrane and solution phase, which in turn enhances the IEC. Nemati et al., reported that the distribution and accessibility of resin particles in the membrane structure enhance ion exchange possibilities [48]. The free spaces (voids and cavities) in the membrane matrix enable the accessibility of the solution to the ion exchange functional groups, which enhances IEC.

The fixed ion concentration (FIC) or the number of functional groups per gram of absorbed water controls the transport pathways of counter ions in the membrane [31]. The FIC can be used as a tool to optimise the relationship between IEC and water content [49]. The CEMs and AEMs demonstrated increasing FIC values as resin loading increased from 1 to 3.5% (Table 3). This is mainly attributed to the water uptake of CEM and AEM membranes, which does not change significantly as resin loading increased from 1 to 3.5% (Table 2). High FIC values are related to better control of pathways and ion movement in the membrane, which enhances membrane selectivity [50,51].

The CEMs possessed lower IEC and FIC values than AEM at the same resin loading. The lower values could be due to the reduction in effective cationic exchange groups in the membranes because of the interactions among the sulfonic acid group of the cation exchange resin and the ortho position hydrogen of the aromatic ring of the PES polymer chain [41,42]. The IEC values (Table 3) indicated that the ion exchange resin particles retained their ion exchange capabilities when incorporated into the membrane matrices. The IEC results obtained for both the AEM and CEM were lower (IEC from 0.06 to 1 meq/g^−1^) than those reported in the literature [30,45]. One explanation for the low IEC of AEMs and CEMs values is the relatively low (1–3.5 wt %) ion exchange resin loaded in the membranes. Previous studies reported the ion exchange resin loadings of 40 to 60 wt % and concluded that increasing the wt % of ion exchange resin in the membrane increases the resulting IEC [18,22,52].

#### 3.1.6. Membrane Potential, Transport Number and Permselectivity

Membrane potential is the electric field that develops when a membrane separates electrolyte solutions of different concentrations [51]. The bare PES membrane had no membrane potential since they do not contain ion exchange resin. Additionally, the CEMs’ and AEMs’ membrane potentials increased with increasing resin loading from 6.44 to 18.97 mV and from 7.64 to 26.0 mV, respectively (Table 3). Membrane potential depends on the surface charge density [50]. In this work, the increase in membrane potential is due to the increasing concentration of charged ion exchange resin particles in the membrane matrix [28]. In addition, increasing the resin loading resulted in uniformly distributed resin particles and additional conducting regions, which, in turn, improves electrochemical properties [53]. Zhang et al. (2015) reported that the distribution and uniformity of the fix charges within the bulk polymer enhance the membrane potential. The SEM images (Figure 1 and Figure 2) showed a uniform distribution of resin particles of the CEMs and AEMs. The membrane potential of AEM was higher than that of CEM, which is due to the decrease in accessibility of cation exchange functional groups in the membrane matrix [54]. The coverage of cation exchange resin by PES polymer due to interaction between the sulfonic acid group of the cation exchange resin and ortho hydrogen of the aromatic ring of the PES [41].

The transport number and permselectivity of the prepared membranes relate to the energy required for the ion exchange process and ionic selectivity, respectively. Transport numbers and permselectivity values followed a similar trend to IEC, FIC, and membrane potential. The transport number of CEM increased from 0.56 to 0.66 with increasing resin content from 1 to 3.5 wt %. Additionally, the AEMs transport number increased from 0.57 to 0.72 with increasing resin content (Table 3). The transport number is the current carried by the ionic group (cation or anion) in an ion exchange membrane [42]. Hence, an increase in resin loading enhances the transport number and selective ionic sites within the membrane. Moreover, a high transport number means less energy consumed in the ion exchange process [31]. Previous studies reported transport values greater than 0.9 [30,55]. The transport numbers for the CEM (0.56–0.66) and AEM (0.57–0.72) were lower than 0.9, indicating that the ion exchange process is more energy-intensive. The results are attributed to the high water uptake, which is a consequence of the voids and cavities. The high water uptake dilutes the charge concentration in the polymer and allows more co-ions to pass [31]. The low transport number is also due to the low resin loading (1 to 3.5 wt %) of CEM and AEM compared to previous literature of 40 to 60 wt % loading [22,28,56]. Membrane permselectivity refers to the transportation of specific species while the passage of other species is restricted. The permselectivity of CEM and AEM increased from 0.26 to 0.44 and from 0.28 to 0.54, respectively, with increasing resin loading. The permselectivity CEM and AEM values are attributed to the increase in the membrane’s fixed ionic concentration which provides ionic channels for the membrane [51,54]. The transport number and permselectivity values (Table 3) of CEMs were lower than AEMs because of the water uptake and IEC. CEM-3.5 membrane water uptake was the highest (65.55%), followed by AEM-3.5 (63.96%), and a high water uptake dilutes the charge concentration of the membrane and hence lower transport number. In addition, the increase in water uptake increases the flow of co-ions and decreases the selectivity [57]. Another possible reason may be the reduction in effective cationic exchange groups in the membranes because of the interactions among the sulfonic acid group of the cation exchange resin and the ortho position hydrogen of the aromatic ring of the PES polymer chain [42]. Thus, the combination of very lower IEC value and high water uptake reduces ionic channels, and, in turn, selectivity.

### 3.2. Membrane Application

#### 3.2.1. Pure Water Flux

The obtained pure water flux value for the bare PES membrane was 8.71 L·m^−2^·h^−1^ (Table 4). The CEMs’ and AEMs’ water fluxes were higher than that of the bare PES membrane and increased with increasing resin content from 1 wt % to 3.5 wt %. For CEMs, the water flux increased from 10.78 L·m^−2^·h^−1^ to 14.90 L·m^−2^·h^−1^ and the AEMs from 11.58 L·m^−2^·h^−1^ to 17.79 L·m^−2^·h^−1^. These results are due to the water uptake, which is related to macro voids observed on the SEM images (Figure 2) that are water channels for water to be transported through the membrane. In addition, the excessive presence of resins might have created preferential water channels at the interface of resin particles and polymer matrix, so the more resins, the greater the increase in water flux. The bare PES gave a lower water uptake than the UF heterogenous IEMs because of its hydrophobic nature with lower water uptake value. The AEMs showed higher pure water fluxes than the CEMs. The differences in the water flux may be since AEMs are more hydrophilic than CEMs (Table 2). Thus, the increase in hydrophilicity attracts more water molecules inside the membrane matrix and causes the water to pass through the membrane, which enhances water permeability [58].

#### 3.2.2. Salt Rejection Studies

**Figure 4:**
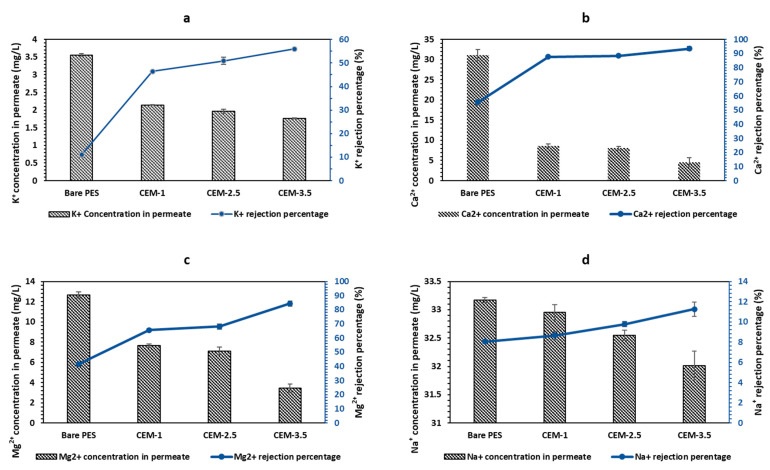
Cation rejection percentage of bare PES and CEMs at 0.5 bars in a dead-end filtration system and concentration value of cation in permeate: (**a**) Na^+^, (**b**) K^+^, (**c**) Mg^2+^ and (**d**) Ca^2+^.

Salt rejection tests by bare PES membrane, CEMs and AEMs were studied using synthetic groundwater. For the CEM, the CEM-3.5 membrane with the highest resin loading had the highest rejection of Na^+^, K^+^, Mg^2+^, and Ca^2+^, while the bare PES membrane had the lowest cation rejection (Figure 4a–d). The highest rejection was observed for Ca^2+^ (93.51%), while the maximum Na^+^, K^+^ and Mg^2+^ rejection values attained by CEM-3.5 membranes were 11.25%, 56.02% and 85.43%, respectively. The feed concentrations of Na^+^ (36.07 mg/L), K^+^ (4.10 mg/L), Mg^2+^ (22.12 mg/L) and Ca^2+^ (70.10 mg/L) after passing through CEM-3.5 were reduced to 32.02 ± 0.25, 1.76 ± 0.01, 3.46 ± 0.40 and 4.54 ± 1.10, respectively, and are shown in Figure 4. The results indicate that the cation rejections are due to the ion exchange mechanism. Hence, the cations’ rejection percentages increased with increasing ion exchange resins. However, cation rejection is observed for the bare PES membrane, which may be due to mere adhesion [59]. The results also indicated that the CEMs have a higher selectivity towards divalent over monovalent ions. The greater rejection of divalent cations by cation exchange membranes has been linked to the higher ionic Stokes radius of divalent cations over monovalent cations [1,20,60]. Thus, ions with smaller ionic Stokes radius are preferentially transported through the membrane [61,62]. According to Luo and Wessling, (2018), the general transport order through CEMs fixed with sulfonic acid groups is: Ba^2+^ > Sr^2+^ > Ca^2+^ > Mg^2+^ > and K^+^ > Na^+^ > Li^+^. Likewise, in this study, the Ca^2+^ with the biggest Stokes radius was retained the most, and the K^+^ with a smaller Stokes radius was less retained.

**Figure 5:**
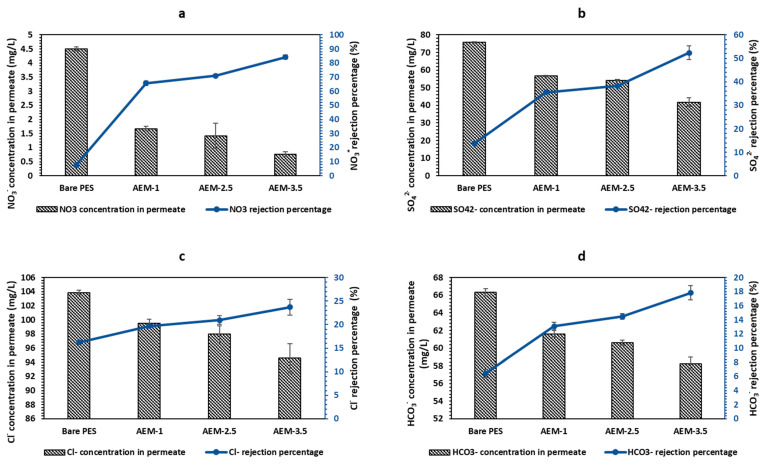
Anion rejection percentage of bare PES and AEMs at 0.5 bars in a dead-end filtration system and concentration value of anion in permeate: (**a**) HCO_3_^−^, (**b**) Cl^−^, (**c**) NO_3_^−^ and (**d**) SO_4_^2^.

Similarly, rejection of anions by AEMs indicated that the AEM-3.5 membrane had the highest percentage of rejection for anions (Figure 5a–d). As with the CEM, an increase in the number of resin particles within the membrane matrix improved rejection. Bare PES membrane had the lowest rejection values for NO_3_^−^, SO_4_^2−^ and Cl^−^. The rejection of anions by the bare PES membrane is attributed to physical adhesion and possible electrostatic interactions [63]. AEM-3.5 membrane had the highest rejection rates, with 84.21% rejection of NO_3_^−^, while SO_4_^2−^, Cl^−^ and HCO_3_^−^ were rejected by 52.36%, 23.71% and 17.83%, respectively. Figure 5 also shows the concentration of anion after filtration and AEM-3.5 has reduced HCO_3_^−^ (70, 90 mg/L), Cl^−^ (123, 94 mg/L), NO_3_^−^ (4.88 mg/L) and SO_4_^2−^ (87.79 mg/L) to 58.26 ± 0.73, 94.56 ± 2.09, 0.77 ± 0.08 and 41.82 ± 2.56, respectively. The higher rejection of NO_3_^−^ can be attributed to the use of Amberlite IRA 900 as the source of anion exchange sites that are nitrate-selective [64]. The order of affinity for Amberlite IRA 900 has been reported as NO_3_^−^ > SO_4_^2−^ > Cl^−^ > HCO_3_^−^ [65]. As a result, the NO_3_^−^ ion was retained the most, then SO_4_^2−^, due to its bigger ionic radius compared to Cl^−^ and HCO_3_^−^ ions. Based on the results, the primary mechanism of rejection of anion was by the ion exchange mechanism.

The above results indicate that the prepared ultrafiltration heterogeneous IEMs underwent ion exchange during filtration. The untreated groundwater TDS level (410 mg/L) was reduced after filtration through the AEM-3.5 and CEM-3.5 to a desirable amount of 237.19 mg/L. A TDS level of less than 300 mg/L in drinking water is considered excellent in terms of improving water palatability and reducing excessive scaling in distribution systems and household appliances (such as water heaters and boilers) [66]. The membranes with highest resin loading gave the best salt rejection percentage, which is associated with the highest IEC, membrane potential, transport number and permselectivity values, and uniform resin distribution. The results clearly show that salt rejections were a combination of mechanisms since the bare PES was able to reject some salts. The mechanism of removal is not limited to charge interactions induced by the resins, but also other interactions such as physical adhesions, inherent membrane charge, which was aimed for this membrane. However, the charge interactions will dominate the removal mechanism.

## 4. Conclusions

The ion exchange ultrafiltration membranes with dual functionality, which includes filtration and ion exchange process, were achieved using the phase inversion method. It was established in this study that the number of resin particles changed the morphology, electrochemical properties, and membrane separation performance. The SEM analysis clearly showed that the polymer and resins were homogeneously mixed. Additionally, increasing resin loading indicated more resin particles per unit area. It was found that the higher resin ratio in the casting solution led to an increase in water content, surface hydrophilicity, ion exchange capacity and fixed ion concentration for the custom-made heterogeneous CEMs and AEMs membranes. Membrane potential, transport number, and permselectivity also increased with the increasing resin ratio loading because of the increased surface charge. The AEMs showed better electrochemical properties than CEMs, which was due to the cation exchange having electrophilic interactions with the PES polymer binder.

The separation performances of the custom-made UF IEMs against synthetic groundwater improved with increasing resins content. The enhanced salt rejections correspond to the increased IEC with increasing resin content. The CEM, cation order of transportation was based on stokes radius size, with the smaller being easily transported through the membrane. Thus, the divalent ions were retained more than monovalent ions. The order of retention by the prepared CEMs was K^+^ > Mg^2+^ > Ca^2+^. The AEMs had a different order of retention, which was attributed to the fact that the Amberlite IRA 900 resins were nitrate selective. The order of retention by the prepared AEMs was Cl^−^ > SO_4_^2−^ > NO_3_^−^. Most importantly, this study has demonstrated that the prepared ultrafiltration heterogeneous ion exchange membranes can exchange ions in the water matrix under low-pressure filtration conditions. The total dissolved solids in groundwater were reduced to a desirable amount of less than 300 mg/L and the result is known to improve water palatability and reduce the excessive scaling ability.

## Figures and Tables

**Table 1:** Casting mixture composition of the prepared membranes.

Membrane Type	Sample	PES (wt %)	PEG (wt %)	Resin Powder (wt %)	NMP (wt %)	Membrane Thickness (µm)
Bare PES membrane	PES	15	2	0	83	190.7
Cation exchange membrane (CEM)	CEM-1	15	2	1	82	197.5
	CEM-2.5	15	2	2.5	80.5	199.6
	CEM-3.5	15	2	3.5	79.5	216.8
Anion exchange membrane (AEM)	AEM-1	15	2	1	82	207.6
	AEM-2.5	15	2	2.5	80.5	220.8
	AEM-3.5	15	2	3.5	79.5	223.2

**Table 2:** The mean surface roughness (Sa), root mean square roughness (Sq), contact angle and water uptake of the prepared bare PES membrane, CEMs and AEMs.

Membrane Type	*Sa* (nm)	*Sq* (nm)	Contact Angle (°)	Water Uptake (%)
Bare PES	471.107	584.916	88.89 ± 4.34	58.89 ± 1.23
CEM-1	42.1155	68.6434	86.13 ± 3.10	61.20 ± 0.50
CEM-2.5	102.084	149.473	84.10 ± 2.05	62.79 ± 0.80
CEM-3.5	317.252	365.124	76.26 ± 2.68	65.55 ± 0.52
AEM-1	68.5571	99.1441	88.02 ± 2.27	63.44 ± 1.45
AEM-2.5	153.511	189.011	73.56 ± 4.62	63.58 ± 0.53
AEM-3.5	295.952	354.847	74.49 ± 4.50	63.96 ± 0.32

**Table 3:** Ion exchange capacity (IEC), FIC, membrane potential, transport number and permselectivity of the prepared bare PES membrane, CEMs and AEMs.

Membrane Type	IEC (meq·g^−1^)	FIC (meq·g^−1^)	Membrane Potential (mV)	Transport Number (t)	Permselectivity (Ps)
Bare PES	0	0	0.08 ± 0.02	0.50 ± 0.0001	0.17 ± 0.0002
CEM-1	0.06 ± 0.003	0.10 ± 0.004	6.44 ± 0.06	0.56 ± 0.0001	0.26 ± 0.001
CEM-2.5	0.31 ± 0.04	0.50 ± 0.07	13.67 ± 0.12	0.62 ± 0.001	0.37 ± 0.002
CEM-3.5	0.58 ± 0.07	0.90 ± 0.12	18.97 ± 0.21	0.66 ± 0.002	0.44 ± 0.003
AEM-1	0.5 ± 0.04	0.80 ± 0.04	7.64 ± 0.15	0.57 ± 0.001	0.28 ± 0.002
AEM-2.5	0.8 ± 0.06	1.26 ± 0.09	19.5 ± 0.32	0.67 ± 0.003	0.45 ± 0.004
AEM-3.5	1 ± 0.09	1.56 ± 0.14	26.0 ± 0.10	0.72 ± 0.001	0.54 ± 0.001

**Table 4:** Pure water flux of the prepared PES membrane, CEMs and AEMs at 0.5 bars in a dead-end filtration system.

Membrane Type	Pure Water Flux (L·m^−2^·h^−1^)
Bare PES	8.71 ± 0.40
CEM-1	10.78 ± 0.75
CEM-2.5	12.50 ± 0.48
CEM-3.5	14.90 ± 0.45
AEM-1	11.58 ± 0.35
AEM-2.5	14.93 ± 0.20
AEM-3.5	17.79 ± 0.29

## Data Availability

The data presented in this study are available on request from the corresponding author. The data are not publicly available because it forms part of an ongoing study that is yet to be concluded.

## Supplementary Materials

The following supporting information can be downloaded at: https://www.mdpi.com/article/10.3390/membranes12080736/s1, Figure S1: The 3D surface profiles of the bare PES and CEMs and AEMs with different ion exchange resins content: (a) bare PES, (b) CEM-1, (c) CEM-2.5, (d) CEM-3.5, (e) AEM-1, (f) AEM-2.5 and (g) AEM-3.5.
