# Supplementary material for: Resin-Loaded Heterogeneous Polyether Sulfone Ion Exchange Membranes for Saline Groundwater Treatment
Source: Membranes (Basel). 2022 Jul 27;12(8):736. doi: 10.3390/membranes12080736 (PMC9416794; doi:10.3390/membranes12080736)
Supplement: Supplementary file 1 [file membranes-12-00736-s001.zip › membranes-1745613-supplementary.pdf]

membranes

Supplementary material for:

Resin-loaded (Enabled/Anchored) Heterogeneous Polyether Sulfone Ion Exchange Membranes for Saline Groundwater Treatment

Fulufhelo Mudau [1], Machawe Motsa [1], Francis Hassard [2] and Lueta-Ann de Kock [1,*]

[1] Institute for Nanotechnology and Water Sustainability, College of Science, Engineering and Technology, University of South Africa, 1709 Johannesburg, South Africa; fulufhelohope86@gmail.com (F.M.); motsamm@unisa.ac.za (M.M.)
[2] Cranfield University, College Way, MK43 0AL Bedford, UK; francis.hassard@cranfield.ac.uk
* Correspondence: dkockla@unisa.ac.za; Tel.: +27-(0)-11-670-9330

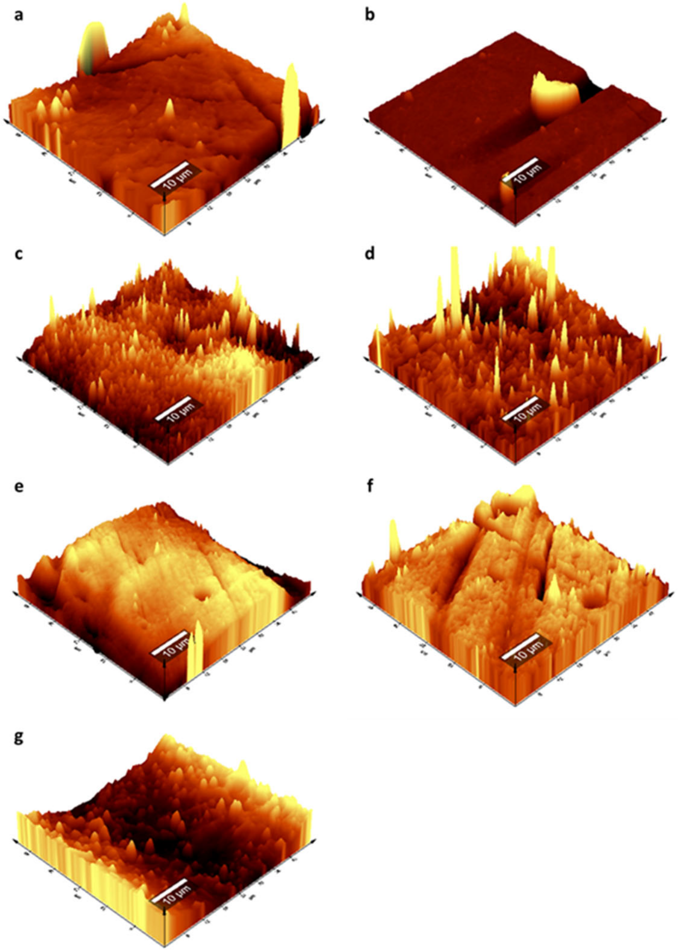

Figure S1. The 3D surface profiles of the bare PES and CEMs and AEMs with different ion exchange resins content: (**a**) bare PES, (**b**) CEM-1, (**c**) CEM-2.5, (**d**) CEM-3.5, (**e**) AEM-1, (**f**) AEM-2.5 and (**g**) AEM-3.5.
